# Epstein–Barr Virus and Hemophagocytic Lymphohistiocytosis

**DOI:** 10.3389/fimmu.2017.01902

**Published:** 2018-01-08

**Authors:** Rebecca A. Marsh

**Affiliations:** ^1^Division of Bone Marrow Transplantation and Immune Deficiency, Cancer and Blood Diseases Institute, Cincinnati Children’s Hospital Medical Center, Cincinnati, OH, United States

**Keywords:** Epstein–Barr virus, hemophagocytic lymphohistiocytosis, primary immunodeficiency, X-linked Lymphoproliferative Disease, Mononucleosis

## Abstract

Epstein–Barr virus (EBV) is a ubiquitous virus that infects nearly all people worldwide without serious sequela. However, for patients who have genetic diseases which predispose them to the development of hemophagocytic lymphohistiocytosis (HLH), EBV infection is a life-threatening problem. As a part of a themed collection of articles on EBV infection and human primary immune deficiencies, we will review key concepts related to the understanding and treatment of HLH.

## Introduction

Epstein–Barr virus (EBV) is a ubiquitous virus that infects nearly all people worldwide without serious sequela (1). However, EBV can cause serious disease complications in patients with primary immune deficiencies. In particular, for patients who have genetic diseases which predispose them to the development of hemophagocytic lymphohistiocytosis (HLH), EBV infection is often an immediately life-threatening problem due to the development of HLH. HLH is a syndrome of severe, life-threatening hyperinflammation (discussed below). Though it is difficult to quantify the association of EBV with HLH in North America and Europe, it is generally agreed that EBV is the most common infection observed to be associated with HLH. In one U.S. series, EBV was associated with HLH in approximately 1/3 of patients (2). For somewhat unclear reasons, EBV is highly associated with HLH in Asia, were it has been observed to be associated with HLH in almost 3/4 of patients in one report (3). It is important to note that HLH also develops in patients with genetic HLH in response to many other infections besides EBV, and it is also common for HLH to develop in these patients without an identified infectious trigger. Patients with X-linked lymphoproliferative disease type 1 (XLP1) are an exception to this statement. XLP1 is caused by mutations in *SH2D1A* (4–6), and HLH is these patients is nearly exclusively associated with EBV.

## When to Suspect HLH in Patients with EBV

Hemophagocytic lymphohistiocytosis should be suspected when any of the characteristic signs and symptoms of HLH are present (Table 1), which include fever, splenomegaly, blood cytopenias, hepatitis and/or hepatomegaly, coagulopathy, central nervous system disturbances, and other more rare complications. However, the distinction between HLH and primary EBV infection can be very difficult, as patients with primary EBV infection may develop some of the hallmarks of HLH as part of natural infection. The timing and severity of these manifestations can help distinguish routine EBV infection from HLH complicating EBV infection. Most patients with routine EBV infection are non-toxic appearing. Fever typically dissipates over time, and splenomegaly should gradually improve. Thrombocytopenia develops in up to approximately 50% of patients, and neutropenia develops in up to 80% of patients with EBV, but cytopenias are generally mild and largely resolve by 4 weeks following infection (7). Mild hepatitis is common with EBV infection and develops in 50–80% of cases, but elevations of liver enzymes are generally mild and improve within a few weeks (7). Jaundice is observed in some cases, but coagulopathy is not typical. By contrast, patients who develop HLH in association with EBV, in association with other triggers, or with spontaneous HLH, are generally ill appearing. Fevers are typically profound and do not improve. Cytopenias are generally life-threatening. Patients usually need transfusion support and are often initially evaluated for hematologic malignancies. Hepatitis can be severe; coagulopathy is common and acute liver failure necessitating a liver transplant can occur. Central nervous system involvement can be profound. Patients may develop focal or global deficits, seizures, and altered mental status. There are not firm clinical diagnostic thresholds that can distinguish routine primary EBV infection from HLH, but good clinical judgment can often help identify patients who are experiencing more severe manifestations of disease and warrant further evaluations for possible HLH. Blood levels of fibrinogen, triglycerides, ferritin, and soluble IL-2 receptor can be measured to help differentiate HLH in appropriate cases, as these markers are typically used to support or refute a diagnosis of HLH.

**Table 1:** Commonly used diagnostic criteria for HLH, adapted from Henter et al. (8).

A diagnosis is consistent with HLH if 5/8 of the below criteria are met, or if the patient has a molecular diagnosis of genetic HLH (including: *PRF1, UNC13D, STX11, STXBP2, RAB27A, LYST, SH2D1A*, or *XIAP/BIRC4*)
1. Fever ≥38.5°C
2. Splenomegaly
3. Cytopenias (affecting at least 2 lineages)
Hemoglobin <9 g/dL (in infants <4 weeks: hemoglobin <10 g/dL)
Platelets <100 × 10^3^/mL
Neutrophils <1 × 10^3^/mL
4. Hypertriglyceridemia (fasting, >265 mg/dL) and/or hypofibrinogenemia (<150 mg/dL)
5. Hemophagocytosis in bone marrow, spleen, lymph nodes, liver, or other tissue
6. Low or absent NK cell activity
7. Ferritin >500 ng/mL
8. Elevated sCD25 (soluble IL-2 receptor): >2,400 U/mL or elevated based on the laboratory-defined normal range

## Diagnosing HLH

It is important to first recognize that HLH is a hyperinflammatory *syndrome*, which is *clinically diagnosed*. A clinical diagnosis of HLH should be suspected in patients with a variety of hyperinflammatory clinical presentations, such as patients who seem to be having a hyperinflammatory process in the setting of EBV infection. Most clinicians use the diagnostic criteria developed by the Histiocyte Society for the HLH-1994 and HLH-2004 clinical trials to help establish a clinical diagnosis of HLH (Table 1) (8). The “classic” clinical presentation is that of an infant or young child with unremitting fevers, pancytopenia, and hepatosplenomegaly. The patient may have a rash, jaundice, or bleeding problems. However, HLH can present at any age, and can present with a variety of other clinical manifestations including hepatitis or acute liver failure, or altered levels of consciousness or seizures if there is central nervous system involvement with HLH. Blood levels of the classic inflammatory markers ferritin and soluble IL-2 receptor are typically high in patients with HLH and are used to help make a diagnosis of HLH. Abnormalities in triglycerides (high) and fibrinogen (low) can also be supportive. Recent evidence strongly suggests that quantification of HLA-DR and other phenotypic markers on T cells can help distinguish patients with HLH (9). Of course, there are exceptions to these generalities, especially in the rare cases of isolated CNS disease in patients who lack any systemic illness. Newer markers of interferon gamma pathway activity or inflammasome activation such as CXCL9 and IL-18, respectively, are also starting to gain in use.

While the criteria in Table 1 can be very useful while considering a diagnosis of HLH, they should be considered to be a guideline only. Some patients with HLH lack 5/8 criteria at presentation, or even throughout their clinical course. Particularly, many patients lack hemophagocytosis in marrow or tissue samples. Additionally, the NK cell function assay has been recently found to be inferior to newer diagnostic screening tests for genetic HLH (10) (discussed later). Another shortfall of the criteria are that some patients who meet 5/8 criteria are ultimately found to have disorders other than HLH, and physicians should be careful to consider alternative diagnoses such as malignancies, infections, auto-immune, and rheumatologic diseases (though these problems can of course be complicated by HLH).

## Primary Versus Secondary HLH

Once a clinical diagnosis of HLH is established, it is important to perform proper evaluations to check patients for genetic diseases which cause HLH (discussed below). HLH can be classified as “primary” HLH, in which case a patient has a proven genetic etiology or has repeatedly developed HLH or has a family history which supports that a genetic disease is very likely. These patients are typically infants or young children. HLH in patients who lack a known or strongly suspected genetic etiology can be classified as having “secondary” HLH. Patients with secondary HLH tend to be older, develop HLH in the setting of strong immunologic triggers such as infections (such as EBV) or malignancies, or in the setting of rheumatologic conditions. HLH that occurs in the setting of a rheumatologic disease is often termed macrophage activation syndrome. Sometimes, treating the underlying trigger of HLH in patients with secondary HLH will lead to resolution of HLH, but varying intensities of HLH-directed treatment are often needed.

## Genetics and Defects of HLH

**Figure 1:**
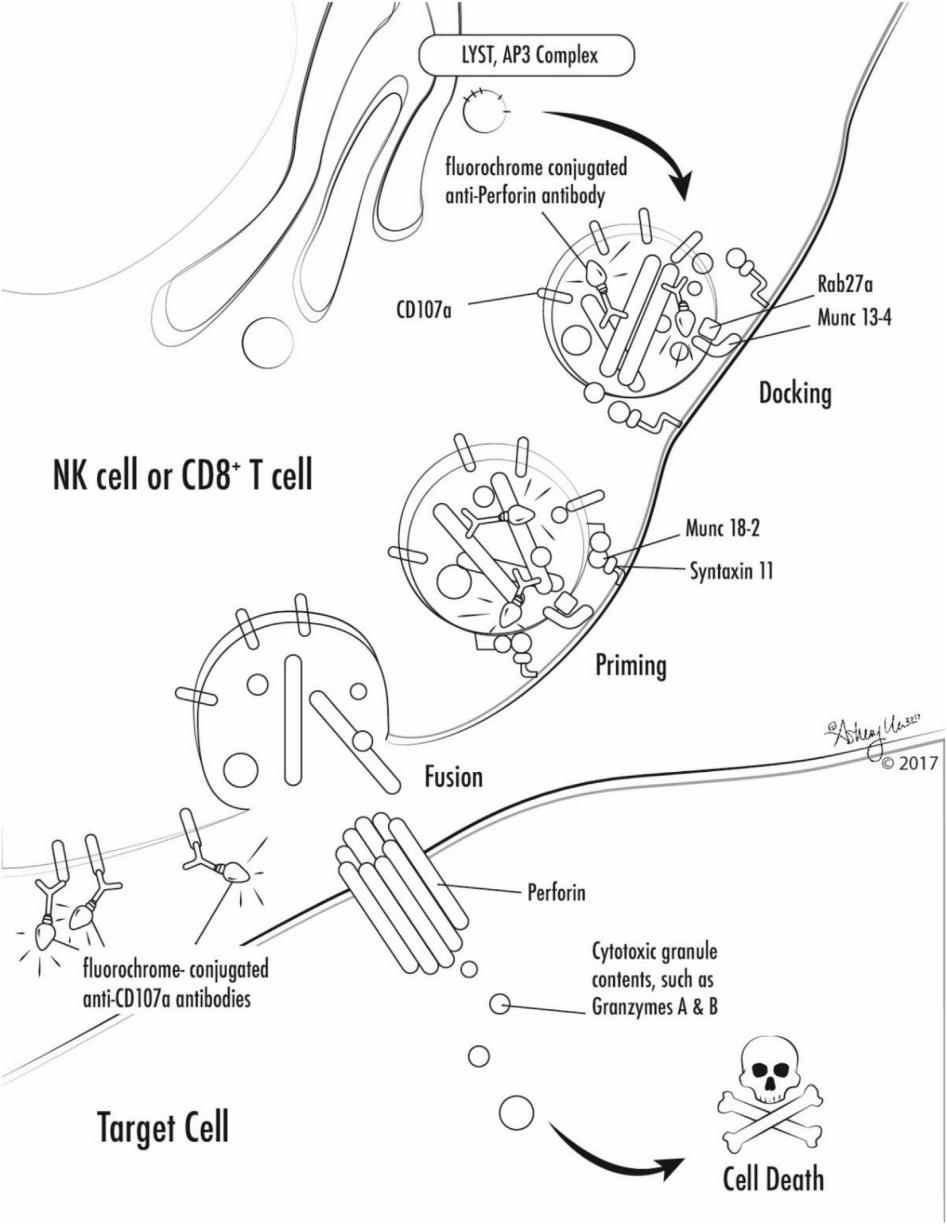
Illustration of involvement of selected primary hemophagocytic lymphohistiocytosis proteins in cytotoxic lymphocyte degranulation and target cell killing. Antibodies against markers used in selected screening diagnostics are also shown (perforin and CD107a).

It was first recognized that HLH could have a hereditary basis in some patients in the 1950s (11, 12). Almost 50 years later, Stepp et al. reported that defects in perforin were responsible for HLH in eight unrelated families (13). The last few decades have seen a tremendous advance in the basic scientific understanding of HLH through the discovery of many additional genetic causes of HLH (Table 2). It is now clear that many genetic causes of HLH essentially cripple cytotoxic lymphocyte granule-mediated cytotoxicity (Figure 1). An exception to this is that patients with X-linked lymphoproliferative disease type 2 (XLP2) due to mutations in *XIAP/BIRC4* appear to have normal cytotoxicity (14, 15), and instead may have dysregulated TNFR and inflammasome function (16). Very recently, activating mutations in *NLRC4* have been found to cause HLH, which also represents another exception to the rule as pathophysiology relates to inflammasome activation.

**Table 2:** Genetic causes of hemophagocytic lymphohistiocytosis (HLH) and associated rapid flow cytometric screening tests.

Disease	Gene	Protein	Rapid screening test
Familial HLH 2	*PRF1*	Perforin	Perforin expression
Familial hemophagocytic lymphohistiocytosis (FHL) 3	*UNC13D*	Munc13-4	CD107a
FHL 4	*STX11*	Syntaxin 11	CD107a
FHL 5	*STXBP2*	Munc18-2	CD107a
X-linked lymphoproliferative disease type 1 (XLP1)	*SH2D1A*	Signaling lymphocytic activation molecule-associated protein (SAP)	SAP expression
X-linked lymphoproliferative disease type 2 (XLP2)	*XIAP/BIRC4*	X-linked inhibitor of apoptosis (XIAP)	XIAP expression, NOD2 Signaling, IL-18 levels
Griscelli syndrome	*RAB27A*	Rab27a	CD107a
Chediak–Higashi syndrome	*LYST*	LYST	CD107a
Hermansky–Pudlak syndrome type 2	*AP3B1*	AP3	CD107a
NLRC4 mutation	*NLRC4*	NLR family, CARD domain-containing protein 4 (NLRC4)	IL-18 levels
CD27 deficiency	*CD27*	CD27	
ITK deficiency	*ITK*	IL-2 Inducible T-Cell Kinase (ITK)	
X-linked immunodeficiency with magnesium defect, Epstein–Barr virus infection, and neoplasia disease (XMEN disease)	*MAGT1*	Magnesium transporter 1 (MAGT1)	

Many genetic forms of HLH are classified together as familial HLH (FHL). FHL2 is due to mutations in *PRF1*. FHL3-5 are due to mutations in *UNC13D, STX11*, and *STXBP2*, respectively (17–20). Related genetic diseases that are associated with HLH and also pigmentary disorders are Griscelli syndrome, due to mutations in *RAB27A*, Chediak–Higashi syndrome, due to mutations in *LYST*, and Hermansky–Pudlak syndrome type 2, due to mutations in *AP3B1* (21–23). Of note, patients with *RAB27A* mutations do not always have abnormal pigmentation. NK cells and T cells from patients with mutations in *UNC13D, STX11, STXBP2, RAB27A, LYST*, and *AP3B1* all fail to degranulate normally, because these proteins are critical for the process of normal cytotoxic granule trafficking, docking, or fusion with the outer cell membrane (Figure 1). The extrusion of cytotoxic granule contents from NK cells and T cells toward their intended target is an important method of elimination of virus infected cells or malignant cells, and also serves to regulate immune homeostasis (24). When the machinery required for this process is broken, intended target cells fail to die and continue to stimulate immune cells, which leads to continued activation and proliferation of immune cells, and a vicious hyperinflammatory cycle ensues.

NK cells and T cells from patients with *PRF1* mutations lack functional perforin. Perforin is normally contained within the cytotoxic granules of NK cells and T cells that were discussed above. Perforin is a unique protein that oligomerizes after release from cytotoxic granules and the complexes create pores in the surface membrane of the intended target cells, which allows cytotoxic granule contents to enter the target cell and ultimately results in target cell death (Figure 1) (25, 26). Lack of functional perforin results in the same pathophysiologic abnormality as other causes of FHL: defective cytotoxic lymphocyte granule-mediated cytotoxicity.

As mentioned above, there are other diseases which are associated with HLH that have different (and perhaps more complicated) mechanisms of disease. XLP1 is caused by mutations in *SH2D1A*, which leads to dysfunctional SLAM-associated protein (SAP) (4–6). SAP is a small SH2 domain-containing protein which is involved in signaling of the signaling lymphocytic activation molecule (SLAM) family of receptors. Lack of normal SAP function leads to several immunologic abnormalities including defective 2B4-mediated cytotoxicity, absence of invariant NKT cell development, defective T cell restimulation-induced cell death, and other humoral and cellular abnormalities (27–30). XLP2 is caused by mutations in X-linked inhibitor of apoptosis (XIAP)/baculoviral inhibitor of apoptosis repeat-containing 4 (BIRC4) (14). Defects in XIAP also lead to several immunologic abnormalities, but cytotoxicity is normal (14, 15). Many cells have an increased susceptibility to cell death, NOD2 signaling is defective, and TNF receptor signaling and inflammasome function are dysregulated (14–16, 31, 32). The development of HLH in these patients is multifactorial, but dysregulation of the NLRP3 inflammasome likely plays a key role in the development of disease. Patients with activating mutations in *NLRC4* also develop HLH, associated with constitutive activation of the NLRC4 inflammasome (33, 34). Thus far, there does not seem to be a strong association with EBV infection, as reported patients have not had an identified trigger of hyperinflammation (33–35). Still other genetic diseases that can be associated with EBV-associated HLH, or chronic active EBV or lymphoproliferative diseases, include ITK deficiency, CD27 deficiency, CD70 deficiency, and magnesium transporter 1 (MAGT1) deficiency which is called X-linked immunodeficiency with magnesium defect, EBV infection, and neoplasia or XMEN disease (36–45).

## Other Manifestations of HLH-Associated Diseases

The FHL disorders are primarily associated with HLH but are often also associated with atypical hyperinflammatory syndromes that lack the full spectrum of HLH, as well as acute liver failure or isolated central nervous system disease in the absence of systemic inflammation. They may uncommonly be associated with atypical chronic active EBV infection, hypogammaglobulinemia, vasculitis, gastrointestinal inflammation, recurrent infections, and very rarely, lymphoproliferative complications (46–48). XLP1 is commonly associated with lymphoma or hypogammaglobulinemia, and has more rare presentations that include aplastic anemia, vasculitis, and gastrointestinal inflammation. XLP2 is associated with a variety of other disease manifestations including atypical/mild HLH-like episodes, inflammatory bowel disease, recurrent infections, hypogammaglobulinemia, uveitis, fistulating skin disease, granulomatous hepatitis, granulomatous, and lymphocytic interstitial lung disease (14, 15, 49–52). CD70 deficiency, CD27 deficiency, ITK deficiency, and MAGT1 all share a strong predisposition to lymphoma.

## Screening Tests for Genetic HLH

While the diagnosis of HLH is a clinical diagnosis based on clinical manifestations and laboratory findings, there are several specialized tests which can quickly screen patients for genetic forms of HLH (Table 2; Figure 1). Flow cytometric screening of perforin expression by NK cells and CD8^+^ T cells serves as a quick screening test for perforin deficiency, and has been found to be highly sensitive (53). A flow cytometric assay to detect abnormal degranulation of NK cells is also available, which quantifies the surface upregulation of CD107a following exposure of NK cells to K562 target cells (or upregulation of CD107a on NK cells or T cells following other appropriate triggers). CD107a is normally expressed within cytotoxic granule membranes, and very little is found on the surface of NK cells or CD8^+^ T Cells at rest. Hence, one can measure CD107a on the surface of NK cells before and following exposure to target cells as a marker of degranulation. This method has been shown to have good diagnostic accuracy for the detection of patients with mutations in the HLH genes associated with abnormal degranulation (*UNC13D, STX11, STXBP2, RAB27A, LYST*, and *AP3B1*) (54). Using both perforin and CD107a testing is more accurate for the identification of patients with genetic forms of HLH compared to traditional NK cell function testing (10). Of note, flow cytometric screening tests are also available to screen patients for XLP1 and XLP2 (55). XLP1 and XLP2 should be considered in male patients with HLH, and even in female patients in whom other genetic causes of HLH have been excluded, due to the observation that females with abnormal skewing of lionization toward XIAP-deficient cells can be symptomatic (56, 57). A functional screen for XIAP deficiency is available via evaluation of NOD2 signaling (58), and IL-18 levels can be helpful as a screening tool for patients with XIAP deficiency or NLRC4 mutations.

## Treatment of HLH, Including EBV–HLH

Once HLH has been diagnosed, therapy should be started as soon as possible. Of note, however, therapy should not be started until complete evaluations for lymphoma and leukemia have been performed. The treatment of HLH generally includes a variety of potent immunosuppressive regimens. In North America and most of Europe, a regimen of dexamethasone and etoposide has been the mainstay of treatment, based on the HLH-1994 and HLH-2004 study protocols (8, 59, 60). The HLH-2004 protocol incorporated the addition of cyclosporine, but there has been no clear benefit related to early administration of cyclosporine (60), and it should be noted that cyclosporine can be associated with notable complications including hypertension, renal injury, and posterior reversible encephalopathy syndrome. CNS HLH is typically treated with targeted therapy if patients are stable enough to undergo lumbar punctures and administration of intrathecal steroids and methotrexate. Approximately 50% of patients can be expected to achieve a complete response, and approximately 30% of patients will experience a partial response (59). The incidence of death prior to allogeneic hematopoietic cell transplant was observed to be 19–27% in the HLH 2004 and 1994 studies, respectively.

In France, an alternative regimen containing steroids and ATG has been used. Seventy-three percent of patients were reported to achieve a complete response with the regimen, and 24% of patients achieved a partial response (61). In general, either approach is appropriate, though there has been more widespread experience with dexamethasone and etoposide treatment. The dexamethasone and etoposide regimen offers avoidance of the risk of severe reactions that can be associated with ATG, and may be less T cell immunosuppressive. ATG offers avoidance of chemotherapy exposure and the risk of marrow suppression associated with etoposide. There has been a recent trial of a Hybrid Immunotherapy approach for HLH^1^ and a European sister trial, but results are not yet available.

Many additional agents have been reported in single patients or small collections of patients. There have been several seemingly beneficial reports of anti-interleukin-1 directed therapies such as anakinra and canakinumab (62–71), and anti-tumor necrosis factor alpha-directed agents such as etanercept and infliximab (72–81), but there are no large data series on which to judge effectiveness. Likewise, plasma exchange has been reported in small numbers of patients, and while some authors report benefit (82, 83), it is difficult to draw conclusions about its effectiveness. More recently, newer agents are under formal investigation for the treatment of HLH. An anti-interferon gamma monoclonal antibody is actively being investigated in the U.S.A. and Europe.^2^ Ruxolitinib is being trialed at a single center in North America for secondary HLH.^3^ Both agents have strong mouse data to support their potential efficacy (84–86). Alemtuzumab is being trialed for up-front therapy in France^4^ and has had reasonable success when used in the salvage setting (below) (87).

For cases of refractory HLH, “salvage” therapy is sometimes needed. Unfortunately, there are very little data on which to base decisions about salvage therapy. The Histiocyte Society Salvage Therapy Working Group recently reviewed the literature, and found that there was evidence for only three agents which had been used in HLH refractory to steroids and either etoposide or ATG: anakinra, ATG, and alemtuzumab, as well as a regimen that combined liposomal doxorubicin with steroids and etoposide (88).

In patients with EBV–HLH, the addition of rituximab can be useful to deplete EBV-harboring B cells and improve HLH. In a retrospective multi-center study, Chellapandian et al. observed that rituximab (given with other HLH therapies) resulted in significant reductions in EBV load within 1 month of use and was also associated with significant decreases in ferritin levels (89).

In addition to these HLH-directed therapies, good supportive care, treatment of underlying triggers, anti-microbial prophylaxis, and close monitoring are usually needed. Anti-fungal prophylaxis, anti-pneumocystis jirovecii prophylaxis, anti-viral prophylaxis, and IVIG replacement during the active treatment period should all be considered. If patients have active virus infections that are associated with HLH such as EBV, CMV, adenovirus, influenza, etc., treatments targeting those infections should be initiated including rituximab for EBV, and anti-viral agents such ganciclovir, cidofovir, oseltamivir, and others as appropriate. The same is true for other infections such as histoplasmosis, tuberculosis, tick-borne diseases, etc. Routine monitoring of laboratory tests such as complete blood counts, liver panels, fibrinogen, and/or coagulation studies should be performed. Weekly or twice weekly monitoring of inflammatory markers such as soluble IL-2 receptor can help with identifying response to therapy or relapse of disease. Monitoring of ferritin can also be helpful, though it is often hindered by changes associated with blood transfusions, and is typically slow to normalize. Newer indicators of pathologic interferon gamma activity such as CXCL9 are gaining favor in use (90), and elevated levels of IL-18 have been found to be a good marker of XLP2/XIAP deficiency and disease activity in those patients (91). Markers of T cell activation such as HLA-DR can also be useful (9). For patients with EBV–HLH, EBV blood polymerase chain reaction (PCR) monitoring can be useful to monitor response to rituximab (89), and also watch for increasing viral loads following rituximab with B cell recovery. Persistently high EBV PCRs following rituximab in the setting of proven B cell depletion can suggest EBV infection of T and/or NK cells.

## Definitive Treatment

For patients with secondary HLH, good medical management and follow-up following HLH resolution is needed. Depending on the underlying trigger of HLH in these patients, they may need indefinite care for management of chronic problems. For patients with proven or strongly suspected primary HLH, hematopoietic cell transplantation (HCT) is generally recommended. Historic outcomes of HCT using myeloablative regimens were poor due to high rates of toxicities that resulted in early deaths. Many groups reported survival of only 45–65% (59, 92–97). More recent reduced intensity conditioning (RIC) approaches have resulted in increased patient survival rates of 75% or higher (2, 98, 99). However, these approaches can be associated with the unique challenge of mixed donor and recipient chimerism, which somewhat limits this success of this approach. Patients with HLH do not require 100% donor chimerism for cure, but risk of HLH relapse increases as donor contribution to hematopoiesis (or cytotoxic lymphocyte development) decreases to less than 20–30% (100). Continued efforts to improve stable donor contribution to hematopoiesis will likely lead to increased success with RIC HCT approaches.

## Conclusion

Hemophagocytic lymphohistiocytosis in response to EBV or otherwise remains a life-threatening problem for patients with genetic disorders that cause HLH. Discoveries made in recent decades have yielded extraordinary advances in our understanding of HLH. Early recognition and initiation of HLH-directed therapy remain key for patient survival. The next decade promises to yield even further advances in diagnostics and treatment breakthroughs which will continue to improve patient outcomes.

## Author Contributions

The author confirms being the sole contributor of this work and approved it for publication.

## Conflict of Interest Statement

The author declares that the research was conducted in the absence of any commercial or financial relationships that could be construed as a potential conflict of interest. The reviewer TG and handling editor declared their shared affiliation.

## Footnotes

^1^https://clinicaltrials.gov/ct2/show/NCT01104025.

^2^https://clinicaltrials.gov/ct2/show/NCT01818492.

^3^https://clinicaltrials.gov/ct2/show/NCT02400463.

^4^https://clinicaltrials.gov/ct2/show/NCT02472054.
